# Impact of Coronavirus Disease 2019 (COVID-19) Outbreak Quarantine, Isolation, and Lockdown Policies on Mental Health and Suicide

**DOI:** 10.3389/fpsyt.2021.565190

**Published:** 2021-04-16

**Authors:** Balasankar Ganesan, Adel Al-Jumaily, Kenneth N. K. Fong, Palak Prasad, Surendra Kumar Meena, Raymond Kai-Yu Tong

**Affiliations:** ^1^Department of Biomedical Engineering, The Chinese University of Hong Kong, Hong Kong SAR, China; ^2^School of Biomedical Engineering, University of Technology Sydney, Ultimo, NSW, Australia; ^3^School of Science, Edith Cowan University, Perth, WA, Australia; ^4^Department of Rehabilitation Sciences, The Hong Kong Polytechnic University, Hong Kong SAR, China; ^5^Department of Rehabilitation Sciences, Jamia Hamdard University, New Delhi, India; ^6^Department of Occupational Therapy, Mahatma Gandhi Occupational Therapy College, Jaipur, India

**Keywords:** COVID-19, mental health, suicide, quarantine, lockdown, social distancing, isolation

## Abstract

The novel coronavirus disease (COVID-19) pandemic has made a huge impact on people's physical and mental health, and it remains a cause of death for many all over the world. To prevent the spread of coronavirus infection, different types of public health measures (social isolation, quarantine, lockdowns, and curfews) have been imposed by governments. However, mental health experts warn that the prolonged lockdown, quarantine, or isolation will create a “second pandemic” with severe mental health issues and suicides. The quarantined or isolated people may suffer from various issues such as physical inactivity, mental health, economic and social problems. As with the SARS outbreak in 2003, many suicide cases have been reported in connection with this current COVID-19 pandemic lockdown due to various factors such as social stigma, alcohol withdrawal syndrome, fear of COVID infection, loneliness, and other mental health issues. This paper provides an overview of risk factors that can cause suicide and outlines possible solutions to prevent suicide in this current COVID-19 pandemic.

## Introduction

The novel coronavirus disease (COVID-19) is a recently discovered infectious disease that is caused by severe acute respiratory syndrome coronavirus 2 (SARS-CoV-2). COVID-19 was first identified in Wuhan, China in December 2019 (1), and it has since spread rapidly to the entire world. On January 30, 2020, the World Health Organization (WHO) declared it a Public Health Emergency of International Concern (PHEIC) based on the International Health Regulations (2005), and they declared it a pandemic on March 11, 2020 (2). The following clinical symptoms present in COVID-19 patients: fever, cough, fatigue, muscle soreness, headache, diarrhea, and dyspnea. However, on April 1, 2020, China's National Health Commission (NHC) reported that 78% of cases were asymptomatic (3). COVID-19 mainly spreads through the respiratory droplets. Besides, people can also be infected by touching contaminated surfaces where the is virus present and then touching their own mouth, eyes, and nose (4). As of May 20, 2020, 4,761,559 confirmed cases and 317,529 confirmed deaths have been reported from 216 countries (WHO, 2020). The United States of America (USA) is in the number one place among a list of countries most affected by coronavirus. For example, according to a report from the WHO Coronavirus Disease (COVID-19) Dashboard, as of May 20, 2020, 1,477,459 confirmed cases and 89,271 deaths were reported in the USA. In India, the second-most populated country in the world, as of May 20, 2020, a total of 101,139 confirmed cases and 3,163 deaths were reported by the WHO (5). At present, there is no vaccination or any other therapeutic method for COVID-19. Therefore, a number of preventive measures have been taken around the world to prevent the spread of infection, such as quarantine, social isolation, lockdowns, and curfews. In this COVID-19 pandemic, ~2.6 billion people have been quarantined or are in under lockdown around the world (6). Although public health preventive measures have been taken to control the spread of COVID-19 infection, it has still had a huge impact on mental health around the world due to various psychological, social, and economical factors, such as loneliness, social isolation, anxiety, stress, depression, fear of COVID-19 infection, loss of loved ones, alcohol withdrawal syndrome or substance misuse, and loss of employment (7–9). Several studies reported that these above-mentioned factors will or have already increased suicide rates during COVID-19 (8, 10–12). A nationwide survey study reported that 34.1% of the quarantined or isolated people had experienced at least one of the following mental health issues: acute stress, anxiety, depression, and sleep disorders (13), and their study also stated that this likelihood was higher in frontline workers, people with pre-existing mental health issues, and people with chronic physical health disorders. Specifically, a previous study found that suicidal ideation behavior was significantly higher among people with pre-existing mental health disorders than in healthy controls in the COVID-19 pandemic (14). Furthermore, previous literature has stated that COVID-19 pandemic-related suicide rates will in the future range from 1 to 145% based on various prediction modeling studies (12). However, limited studies have been carried out on COVID-19 infection prevention measures (isolation, social isolation, locking, and curfew order) and their impact on mental health. Likewise, as soon as these lockdown policies are implemented, there is no updated and functional suicide monitoring system data on the effect of COVID-19 lockdown and other social distancing measures on mental health and suicide. Therefore, in this study, we briefly reviewed the different types of infectious-preventive measures for better understanding and the psychological impact of infection-preventive measures and risk factors for COVID-19-related suicides (up to May 2020). This study also tried to suggest possible solutions to prevent risk factors for the psychological effects of quarantine or lockdown procedures.

## Types of COVID-19 Preventive Measures

These kinds of preventive measures are not unfamiliar; for example, 40 days of quarantine were imposed in Italy during the 14th century to prevent plague epidemics (15, 16), and, more recently, a quarantine was put in place for severe acute respiratory syndrome (SARS) in 2003 (17). Quarantine is one of the oldest and most effective methods to reduce the spread of communicable diseases, and it separates and restricts people who have been exposed to a contagious disease or who have traveled to an affected region; people may not, however, be infected or might be asymptomatic. For instance, more than 150,000 persons were quarantined at their homes in Taiwan to control the SARS outbreak in 2003. Out of 150,000 quarantined people, only 24 people were infected by SARS-coronavirus (SARS-CoV) infection (18). In the COVID-19 outbreak, most of the countries in the world enforced a compulsory 14 days home quarantine or in a designated place (hotel) for people who have traveled to other countries. Under the policies of quarantine, people are not allowed to move from their home or designated place to meet others or invite visitors to their place of residence. If quarantine measures are implemented by the governments, people need to strictly follow the guidelines of the governments or public health authorities. It can be either voluntary or mandatory and implemented at individual groups or community level (19).

On the other hand, isolation has been imposed on the infected people to protect the non-infected people from those people with a confirmed diagnosis of contagious disease. Isolation and quarantine have been considered extreme forms of social distancing. The infected persons will usually be admitted and isolated in the hospital settings under medical supervision (20). In case of infection with mild symptoms, the infected person will be isolated at home.

Another form of public health measure to prevent the spread of infectious disease from person to person is “social distancing.” The “social distancing” measures are implemented to reduce people's physical interactions with other people in the community. Since COVID-19 transfers from human to human (21), the following guidelines are practiced in the social distancing methods: wearing face masks, maintaining at least 6 feet distance from the other persons, avoiding social gatherings, avoiding handshakes, avoiding crowds at the parks, beaches, restaurants, shops, or any other public or private places, and work from home if possible.

The most extreme type of public health measure is “lockdown.” Lockdown restricts the moment of people from one place to another place, and it can be imposed when enough people have fallen sick due to contagious diseases in specific regions or countries (22, 23). The government may order the shut down of schools, universities, public transports, taxis, railways, domestic and international flights, restaurants, temples, churches, mosques, and movie theatres, but hospitals remain an exception (24, 25).

## Impact of Isolation, Quarantine, and Lockdown on Mental Health and Risk of Suicide

**Figure 1:**
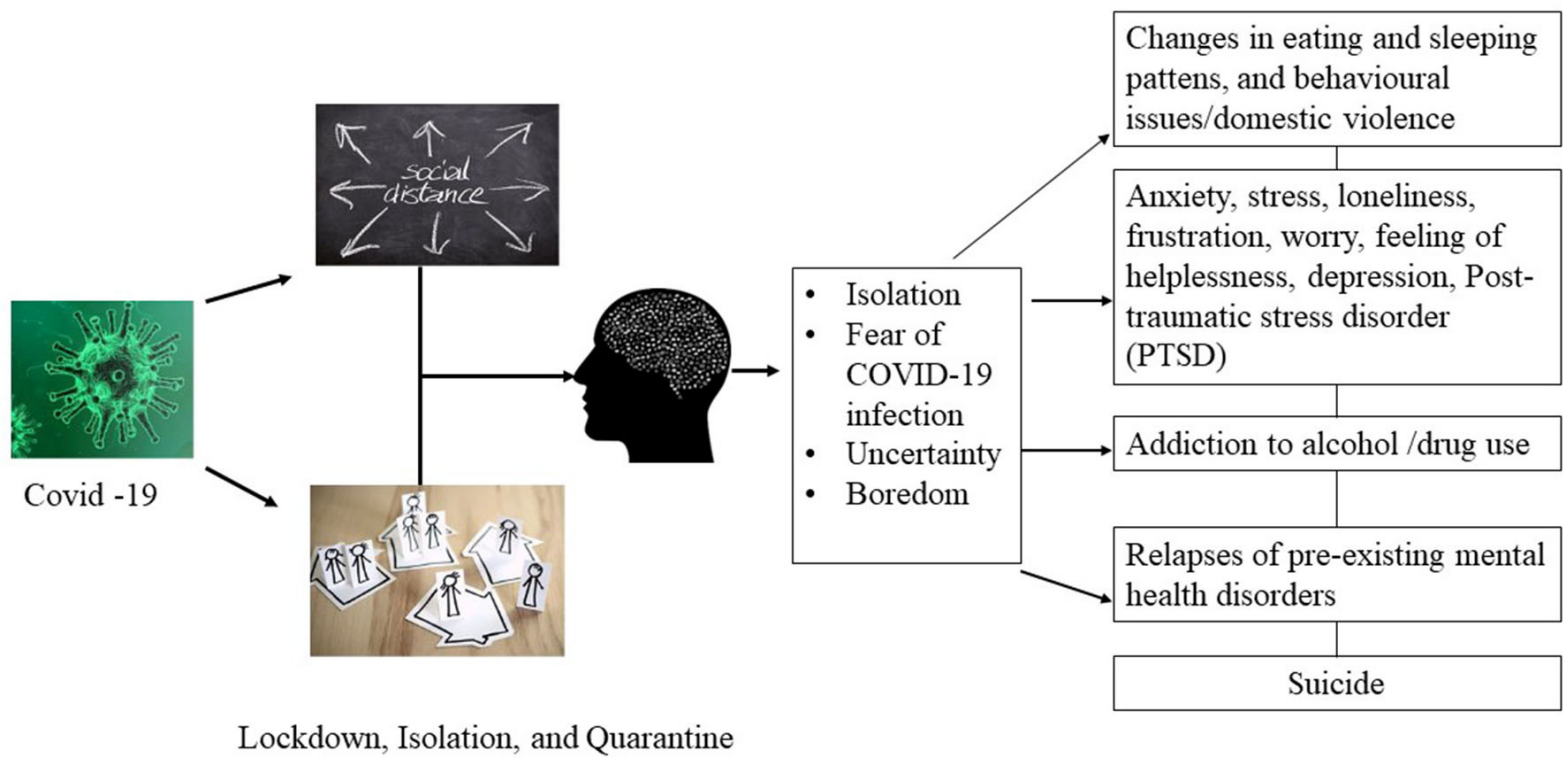
Overview of COVID-19 impact on mental health.

Around the world, the COVID-19 pandemic and lockdown have brought a huge burden to the public, governments, and the economy of the countries. Although quarantines or lockdowns are implemented for a good reason, to prevent the spread of COVID-19, they may cause various adverse effects in the form of physical, psychological, social, and economic consequences. In particular, with social distancing, isolation, and lockdown, people may suffer from very serious psychological issues, such as anxiety, stress, fear, fear-induced overreactive behavior, frustration, guilt, anger, boredom, sadness, worry, nervousness, helplessness, loneliness, insomnia, and depression (26, 27) (Figure 1). A previous study investigated the psychological effects of SARS-quarantined persons in Toronto, Canada, and it was reported that 28.9% of people had a symptom of posttraumatic stress disorder (PTSD), and 31.2% suffered from depressive disorder (28). In extreme cases, social distancing or social isolation may increase the risk of suicide (29), and the following risk factors may trigger suicidal thoughts and ideas: social isolation due to prolonged lockdown, stress, fears of contracting the infection from others, isolated or quarantined individuals with pre-existing mental health issues, loss of employment, financial instability (7, 30, 31), fear of staying in the isolation ward in the hospital, loss of a loved one or missing family members, and feelings of insecurity for the future. At the SARS outbreak in 2003, a higher suicide rate (18.6 per 100,000) was recorded in Hong Kong (32) and most of them were older adults aged 65 or above (37.46/100,000) (33). Loneliness, isolation, anxiety, fear of contracting the virus, and fear of being a burden to their families were thought to be associated with higher suicide rates among older adults during the time of the SARS epidemic in Hong Kong (32, 34). Like the SARS outbreak in 2003, COVID-19 has instilled an uncertainty in people throughout the world, and it has led people to commit suicide, especially, more cases of suicide have been reported in developing countries than in other parts of the world in the early days of lockdown, isolation, and social distance practices (Table 1).

**Table 1:** Impact of Covid 19 and suicides (up to May 2020).

**Case study/No**.	**Age**	**Place**	**Occupation/** **Remarks/Name**	**Gender**	**Risk factors for suicides**	**References**
1	37	Kerala, India	-	M	Alcohol addict/Feeling depressed due to unavailability of liquor	https://news.abplive.com/news/india/coronavirus-lockdown-kerala-man-commits-suicide-after-govt-shuts-liquor-shops-1183618
2	50	Dakshina Kannada, Karnataka district, India	Rubber tapping labourer/ Name: Thomas	M	Alcohol addict/Feeling depressed due to unavailability of liquor	https://www.indiatoday.in/india/story/frustrated-at-not-being-able-to-get-liquor-during-lockdown-2-commit-suicide-in-karnataka-1661060-2020-03-29#
3	70	Karnataka, India	Rubber tapping labourer/Name: Tommy	M	Alcohol addict/Feeling depressed and frustrated due to unavailability of liquor	https://www.indiatoday.in/india/story/frustrated-at-not-being-able-to-get-liquor-during-lockdown-2-commit-suicide-in-karnataka-1661060-2020-03-29#
4	37	Pakistan	Fawad Abbasi	M	Drug Addict/he was suspected for Covid−19 infection and admitted in the Jinnah Postgraduate Medical Centre, Pakistan. But he jumped from the third-floor isolation ward and committed suicide. But his Covid-19 test result showed that he was not infected with the coronavirus	https://www.dawn.com/news/1552729/addict-with-covid-19-symptoms-commits-suicide-at-jpmc
5	-	Chengalpat District, Tamilnadu, India	3 persons: Shivasankar Pradeep and Sivaraman	M	Alcohol addicts: Habitual drinkers/Consumed paint and varnish as an alternative for liquor due to unavailability of liquor	https://www.indiatoday.in/india/story/unable-to-get-liquor-3-men-die-in-tamil-nadu-after-drinking-paint-and-varnish-1663775-2020-04-06
6	27	Tamilnadu, India	Fisherman/Arunpandian	M	Consumed aftershave lotion mixed with soda as a substitute for alcohol	https://www.newindianexpress.com/states/tamil-nadu/2020/apr/04/craving-alcohol-amid-lockdown-two-tn-men-die-after-drinking-aftershave-mixed-with-soda-2125733.html
7	35	Tamilnadu, India	Fisherman/Hasan Mydeen, 35	M	Consumed aftershave with soda as a substitute for alcohol	https://timesofindia.indiatimes.com/city/chennai/three-alcoholics-in-tn-consume-after-shave-lotion-laced-soft-drinks-two-die/articleshow/74981848.cms
8	28	Kerala, India	Name: KC Vigil		Restlessness due to unavailability of liquor to drink	https://bangaloremirror.indiatimes.com/news/india/frustrated-over-not-getting-alcohol-one-more-man-commits-suicide-in-kerala/articleshow/74866273.cms
**MIGRANT WORKER**						
9	22	Kerala, India	Migrant worker/Asif Iqbal Mondal	M	He committed suicide as he did not have money to book the ticket to return to his hometown in Kolkata	https://www.deccanchronicle.com/nation/current-affairs/100520/unable-to-return-home-migrant-labourer-from-bengal-ends-life-in-keral.html
**FEAR & ANXIETY OVER COVID-19**						
10	53	Gurgaon, India	Businessman	M	He committed suicide after his wife tested positive for Covid-19	https://indianexpress.com/article/cities/delhi/gurgaon-54-year-old-commits-suicide-hours-after-wife-tests-positive-for-covid-19-6386371/
11	64	Punjab, India	Name: Santosh Kaur	F	She had a common cold, but she feared it was a corona virus. Then she committed suicide by consuming celphos	http://timesofindia.indiatimes.com/articleshow/75000691.cms?utm_source=contentofinterest&utm_medium=text&utm_campaign=cppst
12	40	Shamli district Uttar Pradesh, India	-	M	He was admitted to the isolation ward on suspicion of having a Covid-19 infection. He committed suicide in the isolation ward, but his Covid-19 test result was negative	https://www.indiatoday.in/india/story/coronavirus-india-suspected-covid-19-patient-committed-suicide-up-hospital-tests-negative-1662942-2020-04-03
13	30	Sidhi district Madhya Pradesh	labourer	M	He committed suicide after being quarantined in Madhya Pradesh, India	https://www.indiatvnews.com/news/india/covid-19-man-commits-suicide-after-being-quarantined-in-mp-610879
14	35	Safdarajung Hospital, NewDelhi in India	Name: Tanveer Singh	M	Covid-19 screening was conducted for all passengers at the Indira Gandhi International Airport, India. During the screening process, Mr. Singh reported that he had headache. Therefore, he was admitted in the isolation ward at the Safdarajung Hospital, New Delhi on suspicion of being infected with coronavirus. Soon after admission in the isolation ward, he jumped from the hospital and committed suicide	https://www.hindustantimes.com/india-news/covid-19-suspected-coronavirus-patient-jumps-to-death-from-hospital-building-soon-after-admission/story-gO78nJO3CIEWAudRtJLRaL.html
15	27	Kenya	-	F	She was sent to 14 days mandatory quarantine at the Kenya Industrial Training Institute (KITI) in Nakuru. According the media report, she committed suicide because she did not like about the conditions of the facility where she was held with other three persons	https://face2faceafrica.com/article/covid-19-south-african-woman-quarantined-in-kenya-commits-suicide
16	36	Mathura, India	Former/Name: Mahendra Singh	M	Due to fear of coronavirus infection, he committed suicide without undergoing any medical test. Because, he assumed himself that he had already been affected by the corona virus infection, so he feared it would affect his family members and villagers	https://newsable.asianetnews.com/uttar-pradesh/cough-and-cold-patient-commit-suicide-in-mathura-up-kpt-q82aun
17	19		Waitress/Name: Emily Owen	F	She committed suicide due to coronavirus fears. In addition, she was previously diagnosed with having high-functioning autism	https://nypost.com/2020/03/25/british-teen-dies-after-suicide-attempt-due-to-coronavirus-fears/
18	60	Ariyalur district, Tamilnadu, India	Name: Narayanasamy (35)	M	Fear of infection/isolation. Mr. Narayanaswamy's village people had informed to the government officials after he got the symptoms of fever. On April 6, he was taken to the Ariyalur government hospital and admitted in the isolation ward. On April 10 (2020), he ended his life himself in the isolation ward. But his COVID-19 infection test results were negative	https://www.newindianexpress.com/states/tamil-nadu/2020/apr/10/elderly-man-in-coronavirus-isolation-ward-commits-suicide-in-ariyalur-general-hospital-2128486.html
**STIGMA OVER CORONAVIRUS**						
19	35	Bibikulam, Madurai, India	Labourer/Name: Mustaffa	M	Mr. Mustaffa was taken to the hospital for Covid-19 testing and test results were negative. Some of his village people took a video when he was taken to the hospital in a small van, then they circulated that video on social media. Moreover, his neighborhoods insisted him to go back to the hospital again. The hospital authority reaffirmed that he was not infected with the corona virus. Unfortunately, he committed suicide on the same day	http://timesofindia.indiatimes.com/articleshow/74939681.cms?utm_source=contentofinterest&utm_medium=text&utm_campaign=cppst
**SUICIDE DUE TO MISSING OF FAMILY MEMBERS**						
20	32	Uttar Pradesh, India	Name: Rakesh Soni	M	According to the media report, Mr. Rakesh Soni committed suicide due to missing his wife as she had gone to her parents' place. Because his wife was stuck at her parents' house due to the implementation of lockdown measures in India	https://www.indiatoday.in/india/story/man-commits-suicide-as-he-missed-wife-in-lockdown-1665050-20
**COVID-19 CASES**						
21	50	Bangalore, India	Autorickshaw driver/Name: Syed Babu	M	Mr. Syed Babu committed suicide by jumping from the coronavirus-ICU ward, due to depression, anxiety, and fear. He was admitted for Covid-19 treatment. He was also diagnosed with hepatitis C infection	https://www.thehindu.com/news/cities/bangalore/bengaluru-patient-ends-life-in-covid-19-ward/article31441997.ece
22	30	Government Medical College and Hospital in Akola, Maharashtra, India	-	M	On April 7, 2020, he was admitted in the isolation ward at the Government Medical College and Hospital in Akola, Maharashtra, India and was tested positive for COVID-19 infection on 10 April, 2020. However, he committed suicide attempt in the isolation ward-bathroom on 11th April 2020 and later he died in the hospital	https://indianexpress.com/article/coronavirus/coronavirus-patient-assam-resident-suicide-akola-maharashtra-hospital-6357770/
**HEATH CARE PROFESSIONALS**						
23	22	Kilpauk Medical College (KMC) hostel, Chennai, India	House surgeon	F	She was found dead at KMC hostel. Hospital authorities confirmed that she was not infected from COVID-19. Police suspected that she may have committed suicide due to stress after being involved in treating covid-19 patients	https://www.indiatoday.in/india/story/medical-student-serving-covid-19-cases-found-dead-in-chennai-hostel-1673188-2020-05-01
24	49	New York City, USA	Emergency Room (ER) Doctor/Name: Dr. Lorna Breen	F	An ER doctor, Dr. Lorna Breen, contracted from Covid-19 during the time of treating the patients. After she recovered from the corona-virus infection, she continued her duty to treat the Covid-infected patients (12-h shift). However, she was sent back her home again by hospital officials and soon later she was again admitted in the University of Virginia hospital for exhaustion. About a week later, she committed suicide after returning home from the hospital	https://www.ndtv.com/world-news/coronavirus-new-york-doctor-lorna-breen-who-treated-covid-19-patients-commits-suicide-2220035https://missoulian.com/news/national/an-er-doctor-who-treated-patients-after-she-recovered-from-covid-19-has-died-by/article_975c2e8f-1597-5a67-9241-79dff309c73f.html
**ECONOMIC ISSUES**						
25	52	Uttar Pradesh	Framer/Name: Rambhavan Shukla	M	Mr. Rambhavan Shukla committed suicide due to non-availability of farmworkers to harvest his wheat crop	https://www.businessinsider.in/india/news/covid-19-lockdown-farmer-commits-suicide-after-no-labourers-to-harvest-crop/articleshow/75106144.cms

### COVID-19 and Alcohol Addiction

Alcohol dependence is a significant risk factor for suicide. It also has high comorbidity with a variety of psychiatric issues such as depression, violent behavior, mood, and anxiety disorders (36). A previous study reported that people with alcohol addiction are 60–120 times more likely to commit suicide than people with no alcohol use disorders (37). On the other hand, after the announcement of a lockdown in India, there have been a number of suicide cases that have reportedly been due to the current unavailability of alcohol in the market. People with alcohol addictions suffer from alcohol withdrawal syndrome when they suddenly stop drinking or significantly reduce their alcohol intake (38). Alcohol withdrawal syndrome is characterized by tremors, insomnia, anxiety, and other physical and mental symptoms (alcohol hallucinosis, alcohol withdrawal seizures, and delirium tremens). According to the India Today newspaper (March 2020), two alcohol-related suicides were reported in the state of Karnataka, India. The first instance was of a 50-year-old man who desperately moved around for few days to acquire liquor before he ended his life due to frustration and alcohol withdrawal syndrome (39). Another person, a 70-year-old man, committed suicide by hanging himself in a tree in the same district of Karnataka due to the non-availability of alcohol. According to News18, seven people committed suicide due to alcohol withdrawal syndromes in the districts of Thrissur, Kochi, Kannur, Kollam, and Thiruvananthapuram, Kerala, India (40, 41), and most of them were younger than 40 years old (42). In contrast, some people with alcohol addiction took to after-shave lotion or paint and varnish as alternative drinks to liquor after liquor shops closed due to lockdown in Tamilnadu, India. Furthermore, it has been reported that three people died by drinking paint and varnish (43), and two people died after consuming after-shave lotion (44).

### COVID- 19 and Social Stigma

Social stigma in the context of health is the extreme social disapproval of a person or group based on a specific disease and its characteristics. Likewise, the COVID-19 outbreak has not only spread fear and anxiety worldwide, it has also fostered various kinds of social stigma, such as discrimination, and racism, and judgmental attitudes toward quarantined or isolated people and people who have traveled to the virus-affected regions or countries. In addition, stigmatized people may be experiencing social rejection or avoidance by others, physical violence, and denial of healthcare services, housing, education, and employment opportunities (45). These types of social stigma not only affect those with the disease, but it also affects their family members, friends, and communities. It can make people afraid to get screened for COVID-19 or any other contagious diseases, and some even take extreme steps, such as suicide or displaying anti-social behavioral issues.

### COVID- 19 and Unemployment: An Economic Issue

Although we save thousands of people from the COVID-19 infection by implementing prolonged lockdown measures, there will be a huge micro-, meso-, and macroeconomic loss to individuals, organizations, and countries. The prolonged lockdown can cause an increasing unemployment rate, and it may drive stress, mental health issues, family issues, intake of more alcohol or substance use, an increase in crime or suicide rates. A recent report from the United Nation Labor Agency stated that this COVID-19 pandemic will have a worse effect on the labor market and may lead to a risk of a 50% loss of the global workforce (46). Furthermore, UN news reported that the lockdown measures will affect almost 2.7 billion workers globally (47). Currently, there are millions of workers who suffer from uncertainties related to food, security, and future life. According to the press release of the International Labor Organization (ILO), on April 29, 2020, the second quarter (Q2/2020) of the global showed that working hours from this year (2020) are expected to be 10.5% lower than the last quarter of 2019 (Q4/2019) due to pandemic lockdown measures. This global working hours damage is estimated to be equivalent to 305 million full-time jobs (48). Specifically, in developing countries such as in India, about 27 million people (age group 20–30 years) lost their jobs in April 2020 (49), and the Centre for Monitoring Indian Economy (CMIE) reported that 84% of the household will be affected by decreased monthly income. Similarly, ~1 million people lost their jobs in Australia (50) and 5.5 million in Canada, which was an increase in the unemployment rate of up to 13%, putting it closer to the unemployment rate in the USA (14%) (51).

A recent report predicts that the worldwide unemployment rate is estimated to be at a maximum of 5.6%, and it will increase the suicide rate to 9,570 per year (52). For example, more than 1,500 were made to the Los Angeles suicide crisis hotline crisis in March 2020 after few weeks of lockdown, and one in five calls were related to suicide (53). All over the world, government officials are also stressed over dealing with the economic fallout of the coronavirus. For example, Mr. Thomas Schaefer, Minister of Finance of Hesse, Germany, recently committed suicide on March 28, 2020, due the COVID-19 crisis (54). Likewise, according to Aman et al. (2020) until now, more than 300 deaths have been reported as non-COVID-19 infection-related deaths following the lockdown in India. Among those deaths, 34 deaths were recorded due to financial hardship and starvation (55), however, these reports have not confirmed how many deaths were suicide related.

### COVID-19 Non-pharmaceutical Interventions and Psychopathological Factors

It is important to identify which factors modulate the mechanism and changes in psychopathology symptoms among the public in the period of the COVID-19 pandemic. A recent Norwegian population-based study reported that people with pre-existing mental health disorders and those who were living alone are affected by loneliness in the period of implementation of social distancing measures, and loneliness was very closely associated with depression and anxiety (56). Another study found that people with pre-existing anxiety-related and mood disorders had more negative impact than those with no mental disorders; anxiety-related disorder groups, in particular, expressed more fears of the socioeconomic impact, xenophobia, fear of danger and contamination, and traumatic stress-related symptoms (57) based on COVID Stress Scales (58). In addition, a psychopathological cross-sectional study found that some specific fear factors (neuroticism, corona phobia, and hypochondriasis) played a role to elevate pandemic-related psychopathology, such as depressive symptoms and generalized anxiety, in the period of lockdown/other preventive measures (59). These prolonged lockdown/other preventive measure factors and the uncertainty of when the COVID-19 crisis is over would also increase the prevalence of post-traumatic stress disorder (PTSD). Recent literature reported that the prevalence of post-traumatic stress disorder (PTSD) during the COVID-19 in Saudi Arabia was 22.63% (PTSD cut-off score), 24.8% (criteria), and 19.6% (combined). In addition, their study reported that the PTSD prevalence was similar or higher to USA (31.8%), Spain (15.8%,), and Italy (29.5%) than China (2.7–12.8%) (60).

On the other hand, a recent study reported that 37 adolescents and youths, including 14 school-age students, committed suicide in the period of lockdown, based on the report of media news between February 15 to July 6, 2020 (35). The COVID-19 prevention strategies related distress, online class/remote schooling, and examination-related stress to tendencies toward depression, loneliness, and psychological distress. Also, a suicide pact (son and mother) was reported in Bangladesh due to an argument between family members regarding online class (61). Likewise, the elderly population has been the most affected age group due to COVID-19 lockdown in terms of isolation or difficulty in obtaining medical and rehabilitation services for aging-related complications. In this COVID-19 pandemic, older adults have a high risk of infection and death (62). Therefore, the elderly population is more prone to fear, stress, depression, loneliness, and other mental issues.

The COVID-19 social restrictive preventive measures related psychopathological factors, such as stress and anxiety, to an increase in alcohol consumption as a coping mechanism. Regarding the COVID-19 and use of substance or alcohol consumption, previous literature estimated that 75,000 “deaths of despair” may result from COVID-19 pandemic related (stress, isolation, and unemployment) drug and alcohol addiction, and suicide (63, 64).

## Suicide Prevention During and After the COVID-19 Outbreak

Across the globe, there is a huge uncertainty is seen among the public due to the coronavirus pandemic. People are facing difficulties in accessing their basic needs (e.g., food and medical services) as well as employment, their futures, and well-being due to the current scenario of the prolonged restrictions on movement, social distancing, and isolation. Besides, social isolation and loneliness can cause serious public health issues among people whether young, middle-aged, or old, and there is a strong association between these and the development of neurocognitive and mental health disorders as well as heart and autoimmune diseases (65); social isolation and loneliness could create negative health outcomes, such as high blood pressure, heart diseases, disability, a decline of cognitive function, and depression (66).

For example, the National Public Health Group-Well Being Trust and Robert Graham Center for Policy Studies in Family Medicine and Primary Care (US) have estimated that around 75,000 Americans could die because of suicide, alcohol and drug misuse, and as a result of the COVID-19 pandemic (67). Another report from the Brain and Mind Centre at Sydney University, Australia, predicted that the suicide rates will double due to the social and economic consequences of the national lockdown measures, and about 1,500 extra deaths will occur in Australia (68). Therefore, worldwide, mental health and suicide experts warn governments to take immediate action to intervene in mental health issues among the public in the COVID-19 pandemic to avoid suicide-related deaths (69). In this paper, the following possible solutions and public health awareness methods have been discussed to prevent suicide among the public and healthcare workers, and Table 2 shows the impact of quarantine, isolation, lockdown, and social distancing (SWOT analysis).

**Table 2:** SWOT: COVID-19 preventive measures (quarantine, isolation, lockdown, and social distancing) and mental health.

**STRENGTH**	**WEAKNESSES**
**Governments** • Helping to prevent the COVID-19 infection • Using more online technological services including health care sector services • Can use public authorities to serve and control the spread of infection • Can use national Media & TV channel to create the awareness • Using social media for spreading awareness	**Governments policies/orders** • Insufficient hospital facilities and medical equipments, including for general and mental health services • Uncertainty of Economy due to various reasons such as, Disturbed in providing essential services to public due to the closure of public and private transports, Closure of schools, universities, Partially or fully shutdown of factories and industries
**Individuals** • Regular exercise/yoga at home • Learn new skills such as learning new language, arts, painting, cooking, take care of family members • Working from home • Spending more time with family • Learning new online courses	**Individuals** • Poor social interaction and Isolation • Difficult to attend social life such as meeting friends, relatives, or going to bars and social clubs • Reduced monthly income
**OPPORTUNITIES**	**THREATS**
**Governments and public health authorities** • Implementing to control the spreading of COVID-19 infection • Can create more awareness through local and national leaders, social influential people, and religious people • Allotting appropriate funding for health care sectors • Allotting appropriate welfare funding for their citizens to manage the lockdown period • Arranging more mental health screening programs • Using media to create awareness among public to avoid the social stigma	**Governments and public health authorities** • Damage to economy of the country • Political threat by other countries • Shortage of medical equipments and devices • Challenging to save the frontline workers from COVID-19
**Individuals** • Can do regular exercise to improve physical and mental health • Can create awareness to support the frontline workers and discourage the stigma • Being as a responsible citizen and family member to the society • Being a role model to children by stopping to drink alcohol and smoking • Learning new courses	**Individuals** • Difficult to do regular health checkup/follow up for those with pre-existing mental health issues such as schizophrenia, bipolar disorder, and other mental health disorders • Students - No face-to-face classroom experiences and prolonged stay at home • Affected by various psychological issues as anxiety, feeling stressed, fear, fear-induced overreactive behaviour, frustration, guilt, anger, boredom, sadness, worry, nervousness, helplessness, loneliness, insomnia, and depression • Obesity and diabetes • Suicidal thoughts • Domestic violence • Unemployment, Poverty

### Recommendations and Suggestions for Social Stigma

Governments, media, the public, and individuals have an essential role to play to prevent the COVID-19-social stigma against ethnicities, religions, and specific countries. Media in particular should carefully select their topics when covering COVID-19 topics. Topics must be factual and respectful to avoid stigmatization, fear, and anxiety among the public. For example, certain words can be replaced by other words “people who have COVID-19” instead of “COVID-19 cases” and “people who may have COVID-19” instead of “COVID-19 suspects” and “suspected cases” (70). In addition, governments and public authority officials can request that celebrities, religious leaders, and leaders of specific regions spread facts about COVID-19 through social media platforms and TV channels. It would be helpful to reach out to the greatest number of people to reduce stress and anxiety among the public. Unfortunately, most healthcare workers have experienced social stigmatization from working with patients with COVID-19. To prevent the social stigmatization of healthcare professionals, showing support and making statements of gratitude on social media aimed at doctors and healthcare workers would increase recognition of their vital role in the community and reduce stigma.

### Recommendations and Suggestions for Social Distancing, Quarantine, and Isolation

Prolonged social distancing and isolation, can impact both the physical and mental well-being of individuals, including healthy people. To avoid the stress, anxiety, depression, and other serious mental health issues, including suicidal ideas, individuals can attempt activity scheduling, such as reading, listening to music, learning, watching interesting TV programs, regular exercise (e.g., stretching and yoga), and learning a new language, instead of continuously watching live coverage of coronavirus-related news. For example, One World: Together At Home is a special broadcast curated by Lady Gaga in support of healthcare workers on the frontlines of the COVID-19 crisis, and this event also raised $128 million to support vaccine development and local and regional charities (71). Although social distancing requires the maintenance of physical space between people, people can contact their family members and friends by using various social apps like WhatsApp, FaceTime, Viber, Skype, Zoom, and Facebook messenger. In addition, this study suggests providing more online telehealth counseling services to the quarantined and isolated people as well as people who are recovering from coronavirus infection.

### Recommendations for Preventing Alcohol-Addiction-Related Suicides

In this study, we have discussed the number of alcohol-dependence-related deaths during the lockdown period. Most individuals try to consume more alcohol to overcome social isolation, assuming that alcohol consumption will reduce their anxiety and stress during the lockdown period. However, alcohol consumption leads to many physical and mental health problems, including liver- and lung-related diseases, depression, anxiety, suicidal thoughts, and social and behavioral problems. In India, most alcohol-related deaths were associated with substance withdrawal syndrome. Therefore, not only quarantined people but also the individuals who have been admitted for COVID-19 care need to be screened for substance use withdrawal syndrome along with COVID-19 care. Also, there are a number of myths about consuming alcohol and COVID-19. Therefore, we suggest educating individuals and raise their awareness of the effect of alcohol consumption and circulate guidelines for avoiding suicide. We list the following topics as suggestions for content for governments policymakers and media to raise awareness among the public:

- Drinking alcohol will not kill the virus and it will not help you develop immunity/resistance to coronavirus
- Drinking alcohol will not help you cope with stress; in fact, it is likely to cause anxiety, depression, and other mental health disorders and vulnerable behaviors
- Contact the local or national mental health counseling hotlines if you have any signs of alcohol use withdrawal syndrome
- Local governments can support the individuals by providing food, medicine, subsidies, and more telehealth-hotline-based psychological intervention services to protect people from alcohol-related suicide deaths
- Local governments should advertise telehealth-hotline-based psychological intervention services phone numbers through social media, television programs, and social influencers
- Local governments should encourage individuals and family members to do regular yoga and exercises at home in this lockdown period
- Avoid drinking alcohol in front of children to be a role model for the child
- Provide more online telehealth services for alcohol-dependent people with pre-existing mental health issues

### Recommendations and Suggestions for Unemployment, Economic Issues, and Suicide

Current worldwide suicide rates high compared to the previous economic recession of 2008. Recession triggers various kinds of stress and mental health issues among individuals due to unemployment, job loss, loan defaults, and government cuts to welfare and healthcare budgets. These financial difficulties will cause more recession-related mental health issues and suicide. To prevent pandemic-recession-related suicide, mental health experts and researchers have to raise awareness toward the governments and societies about an increasing unemployment rate being a risk factor for suicide. The pandemic is expected to bring about miserable outcomes; therefore, governments should implement programs to secure the basic needs of living for their citizens, waiving or extending loan payments, the healthcare and educational costs, and providing more online mental health services, including for inpatients in hospitals. In addition, the provision of more funding for mental health services and setting up more mental telehealth services in every hospital is necessary to control pandemic-related suicides. A previous study reported that two-thirds of suicides occurred among people who have lost their job within the year, and 50% of people committed suicide while they were employed (72). Therefore, early mental health intervention is essential to prevent suicide among both the working and non-working population now and in the future.

### Recommendations and Suggestions for Supporting Healthcare Professionals

In this pandemic, healthcare professionals are more prone to stress and trauma due to dealing with COVID-19. There are a number of factors that cause this: long working hours, less sleep, isolatiton from one's family for long periods of time and worry about one's family's safety, lack of protective equipment, lack of testing kits, less-experienced new staffs, and facing discrimination and harassment from the public from fears due to working with COVID-19. Therefore, the national and local governments should ensure the safety of their healthcare workers by providing enough personal protective equipment and medical testing kits, issuing ordinances for harassment and discrimination, creating awareness among the public about the COVID-19 infection and the importance of healthcare workers, ensuring appropriate working hours with enough resting hours, and providing mental health services to the healthcare workers. Moreover, governments can ensure adequate compensation and healthcare services for healthcare professionals if they contracted COVID-19.

### Physical Activity for Overall Physical and Mental Health

Physical activities will not only improve physical health but will also improve the psychological well-being of individuals (73–75). Previously, several studies have reported that physical activity can reduce sadness and suicidal intention (76, 77). The World Health Organization has recommended a required amount of physical activity per day or week for all age groups for people to stay healthy at home during the time of COVID-19. For example, adults aged over 18 years should do at least 150 min of physical activity per week (moderate intensity), and at least 60 min of moderate to vigorous physical activities per day has been recommended for children/adolescents between the age of 5 and 17 years. For children aged 1–5 years, should do at least 180 min/day of various types of physical activity (any intensity) (78). Therefore, we suggest that doing regular exercise or yoga at home is essential during this lockdown period to overcome mental health diseases, including suicide, depression, and anxiety disorders, and other chronic physical diseases, such as cerebrovascular diseases, obesity, and diabetes.

### Prevention of Psychopathology During and After Lockdown

Early identification and prevention of causative factors for psychopathology is very essential to avoiding “second-pandemic”-related deaths. COVID-19 pandemic lockdown has been a psychopathological burden and has had a negative impact on the quality of life of the public, including school students and children, various kinds of frontline workers, people with pre-existing mental health issues, and the elderly population. Based on the previous literature, fear of COVID-19 is associated with anxiety, stress, and depression (79, 80). In a severe condition, psychopathological factors can lead to suicidal thoughts and attempts among those with pre-existing mental disorders.

The management of coronavirus fear and other factors is essential to prevent mental health issues, including suicide. The prevention strategies for fear are not only related to infection of COVID-19, the mental health intervention strategies and government policies should also be focused on various other factors, such as fear of contamination or loss of employment, pre-existing mental health issues, awareness of COVID-19 infection, awareness of social distancing and lockdown policies, social media news about the COVID-19, arrangement of online counseling sessions for students through their schools, telepsychiatry for isolated patients to alleviate their fear, anxiety, stress, and depression. In addition, government and mental health officers/hospital/service providers should closely work and monitor the services for people with pre-existing mental health issues. Otherwise, recurrence is more likely to occur in people with bipolar disorder and schizophrenia.

## Strengths and Limitations

In our paper, we provide an overview of risk factors causing suicide and possible solutions to prevent suicide in this current COVID-19 pandemic. However, our study has several limitations. Firstly, our review study was conducted during the period of lockdown, i.e., up to May 2020. Secondly, there was no updated and functional suicide monitoring system data on the subject of implementing the lockdown, quarantine, and other social distancing procedures and their impact on mental health and suicide. Therefore, this study mostly used the search engine “Google” as an electronic database to search and analyze relevant information from the online and other media news.

## Conclusion

The COVID-19 pandemic has traumatized the entire world physically, psychologically, emotionally, socially, and economically following the implementation of forced lockdown measures to prevent the spread of infection. Therefore, the local, regional, and national governments need to act quickly along with mental health providers to make new mental health policies and improve the availability of mental health services for everyone (people with COVID-19 infection, frontline workers, those in quarantine/isolation, people with preexisting mental health issues, students, and older adults) to prevent suicide due to the COVID-19 pandemic.

## Author Contributions

BG and RT conceptualized and designed the study. BG, RT, AA-J, KF, PP, and SM analyzed the literature and wrote, revised, and approved the manuscript.

## Conflict of Interest

The authors declare that the research was conducted in the absence of any commercial or financial relationships that could be construed as a potential conflict of interest.
